# Crystal Gold and Gold Foil

**Published:** 1855-10

**Authors:** A. M. Leslie


### ARTICLE III.

*Crystal Gold and Gold Foil.* By A. M. Leslie, D. D. S. Being a Review of Three Articles, one by Dr. W. H. Dwinelle, Dental Journal, April, 1855; one by Drs. Taft & Watt, Dental Register, July, 1855, and one by Mr. W. R. Abbey, Dental Journal, July, 1855.

[In the following article, some strictures are made upon Piggot's Dental Chemistry, chiefly, it appears, on the ground that Ure's Dictionary was used in the preparation of that work. Of course this Dictionary was employed, and so was every other book that treated of the subjects under consideration. In no other manner could the author have done justice to his readers. None but charlatans pretend, in these days, to write on so many varied topics as that book contains, from individual experience alone. We must also deny to Dr. Leslie even the merit of *discovering* the indebtedness to Ure. That is acknowledged fully in the preface.

In the particular instance cited, however, Dr. Leslie is peculiarly unfortunate. It so happens that Ure's article was used for only a few facts in regard to the antiquity of the art and the practice of the French gold-beaters. The rest of it was written from direct observations in the workshop of a very popular manufacturer of foil. Dr. Piggot visited that establishment, saw the various processes there adopted from the parting to the final dressing of the leaves, and conversed freely with the proprietor, who kindly answered his questions and permitted him to make use of the information so acquired.

Neither did Dr. Piggot make any mistake in confounding *gold leaf* with *gold foil,* nor did he use the latter term to accommodate the description of gold leaf manufacture to the comprehension of dentists, as Dr. Leslie charitably supposes. It was upon the manufacture of *dentist's gold foil* that his observations were made, and in reference to this exclusively that his questions were asked. The other varieties of leaf did not specially concern him, on that occasion, as he was writing for dentists. He observed, from the superficial oxydation of the melted ingots that all of them were slightly alloyed, and on calling the attention of the proprietor to this fact, he was assured that in *all the foil* made in that establishment, *a little alloy* was introduced. One of the reasons assigned for this was, that such a course diminished the extreme adhesiveness of the surfaces of the leaves, and facilitated all operations upon them. It will be seen, therefore, that so far as practical knowledge could be obtained by one not actually engaged in gold beating, the author of Dental Chemistry and Metallurgy has been neither indolent nor inattentive to his duty as a writer.—ED.]

DR. DWINELLE says, "Dr. Leslie is mistaken in assuming that England was the first to make a preparation of sponge gold, Dr. C. T. Jackson, of Boston, discovered a method by which gold could be produced in this form." Dr. D. in his hurry misapprehends my language: my words are, "for a few years back, the attention of the profession has been gradually drawn to the consideration of new methods of preparing gold for plugging teeth, hoped to be superior to the old mode of beating into leaf. England is entitled to the laurels for having taken the initiatory steps, but Columbia is likely to bear off the palm of victory, if victory there be on this field."

Now, what is the evident point in this? Certainly that England, through some of her professional men, (dentists and chemists,) had drawn the attention of the profession to the *probability of preserving teeth* by preparations of gold *other than gold foil.*

I cannot think that Dr. Dwinelle will deny that in this sense the English took the initiative. He cannot surely forget the reports published in the Journal of the efforts making in England to produce good work with sponge gold, and the trials made by some of the profession on this side the Atlantic, of English sponge gold imported by them. He surely cannot, also, overlook the following strong language used by himself in his first paper on sponge gold.

"While in Europe, a little more than a year ago, Mr. John Tomes, surgeon dentist to Middlesex Hospital, London, showed us an article of sponge gold, uniting more durable qualities than we had hitherto any knowledge of. * * * To Mr. Joseph Barling, No. 9 High street, Maidstone, Kent, *is the profession indebted* for this beautiful article, so *full of promise to our profession.*" Dr. D. then proceeds, I think in rather a historical way, to give the results of experiments made by Dr. A. J. Watts, of Utica, N. Y., and introduces him in a measure to the profession, but neither in that paper or any subsequent one, (his letter excepted,) gives Dr. Jackson any credit. The doctor having named Dr. Jackson, should, in justice to him, have been more minute. "Honor to whom honor is due," is a motto I am guided by, and believe the same is true of Dr. D., still I must add, that *had* I made the assumption the Dr. attributes to me, his articles on sponge gold have been well calculated to lead me to it.

Again he says, "Dr. A. J. Watts' interests have materially suffered from the similarity existing between his name and that of Dr. Watt, of Xenia, Ohio. * * * Dr. Leslie, in his manuscript falls into the error of writing Dr. Watt's name *Watts*, and so it is printed in the Journal." I regret this mistake, more on Dr. Watt's account than my own, because *my* error is made the occasion of some strictures he has not merited. It is a mistake others, I believe, have as carelessly made as I.

In justice to Dr. Watt I would say, he had no opportunity to read my report until he saw it in the Journal, and consequently had no chance of setting me right in spelling his name. In future I will endeavor to recollect Watts—Watt.

A portion of the letter is taken up with some reflections on the course of Drs. Taft & Watt in manufacturing for sale crystal gold. Here Dr. D. misapprehends my report or letter. Dr. Taft, in saying he preferred crystals made by amalgamation with mercury, did not cite the patent process as laid down in the Recorder. It was I that made the reference to render his remarks of service to the general reader. Dr. D. refers to the promise of Drs. Taft & Watt to explain their mode of manufacture. This, together with an article on this point in the Dental Register, from the pen of Drs. T. & W., entited, "our position on the gold question," seems to make it proper in me to make a fuller statement of the circumstances originating in said promise, so that if their enemies will "teaze" they need not distort the facts.

The first public announcement we have of their manufacture, is made by the editor of the Dental Register, who acknowledges the receipt of some of their prepared gold. See October No. for 1853, p. 72. In the next (January) number of the Register we have a paper entitled "Crystal gold for filling teeth, by J. Taft, Xenia, O." In an editorial note, in said number, a pledge is given, on their behalf, that they will give the profession the benefit of their experience in preparing, so soon as they complete some experiments they were making for the perfection of the article. "*In the meantime they are supplying the profession as fast as they can, and are anxious that all may test it thoroughly.*

The paper by Dr. Taft, just named, was struck off in pamphlet form, for more general distribution, and copies handed to the members of the Mississippi Valley Association of Dental Surgeons, at its meeting the succeeding month, (February, 1854.) At this meeting, the subject of crystal gold was discussed, and its use very fully argued, by Dr. Taft in particular. The fact that Dr. Taft in his paper had made *no* allusion to the patent mode of Dr. A. J. Watts, of Utica, described in the Dental Recorder for July, 1853, or acknowledged *any* indebtedness to that source, in his remarks at the February meeting; the fact that Dr. Watt, of Xenia, was a lecturer on chemistry, together with my belief of their entire willingness to give to every one, patentee or anti-patentee, all the honor due them, led me to the opinion that possibly they had found another, it might be an improved, mode of making crystal gold, one which they would not patent, but as members of that society would cheerfully impart; I, therefore, prompted by this fairly reached conviction, made bold to ask Dr. Taft, as he finished his argument, if he would be pleased to describe their process. The answer was the one he has been three times reminded of through the press, but not until such time had elapsed as would have enabled him to have made a candid statement of their process. To be humbugged is very provoking. To have A. J. Watts' method passed on us as that of Taft & Watt is impossible if the latter describe their process. This they will not do yet, still they have done something, they have taken a "position." They say our promise had "*no reference to time!*" Indeed, then "the good time coming" we have been hoping for the past year is a delusion.

Drs. Taft & Watt in their new "position," say further, that they have been advised by dentists to withhold their experience until the utility of crystal gold *is settled.* The *argument* of these friends is, that those not chemists would flood the profession with spurious preparations, and thus crystal gold would be condemned through ignorance in its makers, and the doctors say, that *if* this be a correct view (they admit it is plausible) a promise to publish *now* "would be better broken than kept." Ah, indeed, these are sage advisers. How injudicious it must be in the editors of the Journal and Recorder, (I have not full files of the Register and News Letter at hand and cannot speak positively of them,) to spread before those "who fancy themselves chemists," Dr. A. J. Watts' patent mode of making crystal gold. Could you not see the danger there was in it of "flooding the profession with spurious preparations?" Of putting crystal gold to trial on the wrong indictment? Dont you know the ease with which the profession (on account of its gullibility) may be cheated with a "spurious article?" It is to be hoped, that before you do so again, you will consider "our position on the gold question." I would advise Drs. T. & W. that such friendly arguments as they take position on are simple fudge.

Why cannot you trust the profession as safely as you do the students of the Ohio College; you taught them how to make crystal gold. If you were far enough advanced in experiment to teach them, as well as make an article to be sold at six different depots, when it would be sold to *all* who would buy, not "the better class of operators" only, surely the future reputation of crystal gold was not in great danger by the publication of your experience, especially if *your* mode is an improvement.

It wont do for members of the Mississippi Valley Association to plant themselves in "our position on the gold question" and cover themselves with the old nurse's banner "dont teaze." To these gentlemen, especially, we cannot grant so *little* a position. They are not babes, but occupy the ground of full grown men—public professional teachers at that.

Their writings are public property, and they are the last men who should complain of having their harbors sounded. "Dont teaze," forsooth. Those who are in the wrong, most dislike to be teazed with the truth. The truth, I believe, is this: Drs. T. and W., are indebted to Dr. A. J. Watts for all their success in making crystal gold, but they lack the magnanimity to acknowledge it.

I feared this was the case immediately on the delivery of their first lame excuse for withholding their process, was confirmed in it by the failure to make good their promise, and became fully satisfied of it at the meeting of the Mississippi Valley Association of Dentistry, last February by the admission of one of their students in the presence of Dr. T. We were examining some of the crystal gold, when some remarks were made by me as to its manufacturer. The student remarked, "Oh! let him alone, he makes it after the patent, but he is ashamed to say so; he has instructed us all here this winter how to make it." Really, gentlemen, I am *very sorry* you have since written, "Our position on the gold question;" sorry for your own sakes, sorry for the society's sake, with which you are connected, that you did not at once and promptly give credit to the source of your knowledge, be it to whom it may, I care not, I care only to maintain in our society the *principle* of "honor to whom honor is due," to the patentee as much as to the anti-patentee, so far as acknowledgment of talent is concerned. Above this, however, the anti-patent discoverer commands our admiration. From the first, I perceived the danger to arise from holding their mode secret: First, it gave grounds for surmising they were benefited by the patent; secondly, if they use *it*, another's rights were invaded, if it was a valid patent; and, thirdly, the society would be humbugged and its reputation injured by their course. To avoid all this if possible, but at least to guard the society, I, on the *first occasion*, brought them to the test. I can truly say that I do still believe they had experiments under way at the time they stated, by which they *hoped* to make crystal gold on a different mode than the patent one, but also I am compelled to believe the gold they then, and subsequently, exhibited and sold, and may be now making, was and is made essentially after A. J. Watts' patent; a patent so clear that almost any tyro who "fancies himself a chemist" may meet with success equalling theirs, by following strictly said patent.

It is true, as they say, that they have declined, in some instances, to furnish it when ordered, but this is a recent matter I believe, and may be attributed to the "teazing" they got in the Journal by a particular friend of A. J. Watts, viz. Dr. Dwinelle. Since then, they have declined furnishing it to some dental depots. They are even willing to go further than the plausible arguments of their professional friends require; they are willing, not only to withhold their experience, but also their gold, until the practical utility of crystal gold is settled; by that time I verily think the patent will have expired. Continue, gentlemen, in your experiments, and hide yourselves behind your nursery banner "dont teaze." May your next birth be a creature which will bear the light.

Dr. Dwinelle satisfactorily explains the strange position his article, *as printed* in the Journal, April, 1854, and quoted in my report, April, 1855, placed him. The printer, by adding an s after the word acid, led me to the belief, that the Doctor thought he had a better way of purifying gold, than by acids, whereas, he meant to say, that gold often contained foreign metals not acted on by *nitric* acid. I would suggest that had it been printed as written, it would not then have been quite clear that the Doctor referred to the process called parting, as the proper solvent of the nobler metals is, nitro-muriatic *acid*, not acids, so that to say gold contains foreign metals, not acted on by acid, would not be strictly correct. The meaning of Dr. Dwinelle, however, is perfectly correct, and I fully agree with him, that there are other methods superior to cupellation or cementation for refining gold for dental purposes, albeit much room is taken up in our books describing these (except in assaying) obsolete modes. Still I must think, until the Doctor accomplishes something new, that he has a hard task before him, when he proposes to give us a mode of refining gold, by which this metal may *in all cases* be obtained *entirely pure.* Man is finite, and his means are finite. If he can make gold *always* absolutely pure, it would approach the exactness of the infinite. I hope he may accomplish it. I doubt it, however, but will not prejudge his promised article. Most assuredly the Doctor has my sympathy in his suffering from typographical error, as I believe he does with me in the many instances, some of them ludicrous, mostly trifling, but a few of moment I have been obliged to bear. One thing the printers will acquit me of, to wit, complaining; I fear, however, they will charge me with not writing a very fair hand.

Dr. Dwinelle very kindly, no doubt, although spiced with just a little sarcasm, recommends to my perusal, a few pages of Piggot's Dental Chemistry and Metallurgy, embracing methods of refining gold, and gold beating. He says, "Dr Leslie will find much important information on the subject of gold in Piggot's Dental Chemistry, pages 289 to 312, inclusive. He will there find many of his erroneous impressions corrected, not only in reference to the character of gold, but also as regards its manufacture into foil. Dr. Leslie is mistaken when he thinks gold-beaters do not alloy their foil. Many manufacturers practice it constantly, and justify themselves on the ground that it is better. The fact, that some of the most popular foil makers furnish articles of two and even three qualities, I supposed was familiar to every one. Both copper and silver are used in combination with gold for the purpose of producing their varying qualities."

This quotation must make the Doctor himself smile, as he reads it again, when I tell him I served a regular apprenticeship to the art and mystery of gold beating, and also carried on the business on my own account some eight or nine years. I therefore think that I have a right to correct some "erroneous impressions" made on the mind of Dr. Dwinelle, by reading the aforesaid portions of Piggot's Dental Chemistry. I had, I may say, a year ago read the portion pointed out by Dr. D., and much more of said work, and regreted to find that I had many years before read and paid for very similar matter under the name of Ure's Dictionary of Arts and Manufactures.

Dr. Ure in giving an account of how gold is beaten into leaf, from a lack of practical knowledge, he being a chemist, had to depend upon translating a French description of the process. This, although differing some from our mode, is good enough to give the general reader an idea of how the gold leaf is made. From its similarity, this article of Ure's, would seem to have been taken by Dr. Piggot, and transposed somewhat, he introducing the term foil to make it suit better for a dental work, and indicating correctly the tool in which the dentist's foil is generally made; but its being mixed up with the description of the manufacture of gold leaf for *gilding purposes*, which is *always alloyed* and made of many different colors, and degrees of purity, Dr. Dwinelle has been led into the error of charging gold beaters, in general, with alloying their *foil* made for dental purposes; an error into which dentists in general will be apt to fall, and in this way, many more than Dr. Dwinelle will be ready, "most emphatically to endorse the statement of Dr. Leslie, that for want of chemical knowledge, great ignorance prevails respecting the purity of gold foil." They will decide it as he has done, by the books, not by *chemical tests.*

Dr. Dwinelle thinks my report calculated "to do great injustice to some of those to whom the profession are under peculiar obligations; and to misrepresent the actual condition of dental progress." I had hoped that in the July number of the Journal, the truth of this charge would have been attempted to be sustained by the continuance of Dr. Dwinelle's review, as he had purposed in April. He certainly will need to back it up more clearly than he has yet done. That he, in his anxiety to introduce crystal gold favorably to the profession, *did* do great injustice to a class of men the profession and the public are a great deal indebted to, by charging upon them in April, 1854, and in April, 1855, that they purposely make foil from impure gold, with two or three exceptions, one only of which is named, is I think much easier shown.

I think I know the gold beaters as a class as well as any man in the profession can, and of them I would say, that while I believe them possessed of faults as well as professional classes, I do not believe that any of them are (as said before) *fools enough* to alloy gold intended for dentists' foil. They may not be able "to obtain it in *all cases* entirely pure," but I believe they *all* honestly aim to make it *pure*, and the most of those who are employers, possess more chemical knowledge, than Dr. Dwinelle and the profession may credit them with. It would, however, doubtless, benefit them to learn of Dr. D. how they could *always infallibly* make gold pure. We shall wait, we confess, rather impatiently for the Doctor's mode.

Dr. Dwinelle, seemingly still contends that Abbey's *foil* is a proper standard for the color of pure gold: I objected to this, first, that it was variable in color. Dr. D. admits, that a number of years ago "Abbey's foil was quite red." Mr. Wm. R. Abbey in the last (July) No. of the Journal says, that for the last five years, at least, it has not been red. Then, according to both, it was red at one time; both claim it is not so now. But this admits that it has varied. Mr. Abbey, further says, "fine gold has the property (one not generally known) of becoming slightly deeper and darker in color as it is reduced in thickness, the difference in the shade being rendered more distinct by annealing. Thus the thinnest and the thickest foil, (say Nos. 4 and 10,) though made from the *same bar*, and undergoing the same treatment in all respects, *will differ in color* slightly, the thinnest being the deepest or darkest shade. * * * Were the complete foil and the original bar placed in contrast, the *difference in shade would be quite obvious.* I trust I have made clear, how our foil may be uniform in its color in one sense, though at the same time *variable* in another sense, and also why the melted button is a shade paler, than the leaf or two of foil of which it is composed."

I am truly glad Mr. Abbey has written on this subject, because I believe he knows practically what he is speaking about. He clearly admits that in different stages of manufacture Abbey's foil is variable. With all this variability Mr. Abbey strangely insists that his foil is of a *natural genuine fine gold color. Which* number of it does he refer to? No. 4, or No. 10, they differ he admits, or does he mean the foil *before annealing.* He admits that annealing makes "the difference in shade more distinct." I am at a loss to know which of these *three* shades is the *natural one* with him. Can Dr. Dwinelle help me? If he selected *either* one of them, then the other two must be *unnatural,* they must be *artificial.* If he selects the unannealed foil, then Abbey's "complete" foil, the article in the market, cannot be taken as a "standard for all the world." If we take his No. 4, it will condemn No. 10 as one of the "paler foils of the market," *if* Mr. Abbey be right, when he says that fine gold has the property of becoming darker as it is reduced in thickness. This is a strange theory of Mr. Abbey, by which he hopes to explain the variability of his foil.

I have made a large amount of fine gold into foil, and I never observed any change in color as it was reduced in thickness, and I will venture to assert that Mr. Abbey does not find any difference in color to exist between his Nos. 4 and 10, or between these and the bar from which they were made, *before* the foil is annealed, and that the difference in color is a *result* of annealing dependent on the presence of something in the gold, and which is more readily yielded up to the action of the atmosphere by a *thin* body of gold than by a thick one, and *thus* he has the difference in color between different numbers, *if* this difference really can be shown in foil from the *same* bar. There is a much greater likelihood of finding variability of color from a change in the amount of the substance which produces the color present in the gold than there is from the variation in thickness.

I have said that Abbey's foil is of a peculiar red color, and that this color was only present when iron was used in its preparation. Mr. Abbey says, it is true, iron has this effect upon gold, but for the last five years to his knowledge it (Abbey's foil) has not been red. I still assert, that even now, it is of a peculiar *red* color. That the foil as found in the market is not of the "genuine natural color of pure fine gold." That this color *is superficial* and is *redder* than the *cut* surface of a button made from melting it, and also redder than the *bar* from which it was made, or the foil *before annealing.* Mr. Abbey admits that the foil *when completed* is "deeper or darker" than the bar, contrast them, (the foil and bar,) and "the difference in shade would be quite obvious." Now what he terms "deeper or darker" I call red, because it is the most proper term. I cannot say it is yellower than other foils. Orange would not, combined with yellow, produce that color. It is really a blending of *red* with yellow. But, call it what we may, Mr. Abbey admits the difference, but claims the foil is of the natural color. I say the cut surface of the bar or button made from Abbey's foil is nearer a true standard, and is about the color of pure gold, and also of nearly if not all good foil.

It is true, Abbey's foil is not now *as* red as it was before the period designated by Mr. Abbey, or during that spoken of by Dr. Dwinelle, (at which time Mr. Abbey tacitly admits it was caused by the presence of iron, which is the *real fact,*) but it only *now* differs a *degree,* and is a result of the *same agencies* better controlled.

Mr. Abbey says, "hence, if Abbey's foil *is* discolored, or red, (as per Dr. Leslie,) the button formed from melting a leaf or two will partake of the same shade of red." This will be found true in *very red* foil, to some extent. A portion of the button will likely show the red color, but like Abbey's present make of foil, "the shade is rendered more distinct (it having been first made as *thin as foil)* by annealing. Gold, with a *small* amount of iron in it, will not show the deep color by the ordinary annealing of the bar, the ribbon, or the small button. It takes the heat of the muffle applied to the beaten foil *to bring it out,* and *this* is the reason, that, if you melt a leaf or two of Abbey's foil, the red color does not show on the surface of the button, which is paler than the foil. I cannot understand Mr. Abbey's *mode* of reasoning when he says, "if it (Abbey's foil) is *not* discolored, superficially or otherwise, but is, as I assert, natural and genuine in its color, the button will be slightly *paler than the foil.*" The English of this is, if Abbey's gold is not discolored in a certain state, (that of foil,) why it *is* discolored in certain other states, such as in a button, or bar, or even in the form of foil, *before* annealing. Yea, even No. 10 must be discolored, for it is not the same as No. 4 in color. I would reason that if the foil is *not* discolored, the color of the (cut) surface of the button would be *exactly* the same as the foil.

Dr. Dwinelle, perhaps without noticing the change, shifts his ground from Abbey's foil as sold, to Abbey's foil *burnished.* That is, I suppose, he finds a finished burnished plug made of Abbey's gold foil offers no contrast to a button of the same gold burnished. I believe it, for there he has got rid of the superficial coloring by means of the finishing file and stone. I am perfectly willing to look on such a plug or button as a tolerable standard of the color of pure gold, but prefer it unburnished, just as it comes from the stone. You may burnish gold and silver so highly, that they have, what workmen call, a black burnish.

Nothing I have said has, or is, intended to disparage Messrs. Abbey's foil; I believe there is none made which, in good hands, will preserve teeth better. In saying that its deep color could only be obtained "when iron was used in its preparation, and need not be then, if the gold is perfectly washed," I did not intend to charge them with ignorance or inattention in the process of purification. I may venture to say, although I never spoke with them, that I *know* they are neither ignorant in, or inattentive to, every step best calculated to produce the results they have in view.

Mr. Abbey misconstrues my use of the word "superficial." I said the *color* was only superficial; he says, the deep color "arises from no artificial or superficial *process*." I grant that he is guided by science, but I most positively affirm, that the deep color of his foil is the result of an *artificial* process, and that this color is obtained *only* when iron, (say as a proto-sulphate,) is used in the precipitation of the nitro-muriatic solution of gold. The *perfect* washing of this precipitate, (which Mr. Abbey, I am sure, can accomplish as well as any one if he wished it,) will generally produce gold, which, when beating out into foil, will be of the same color *after* annealing as before, and "were the complete foil and the original bar placed in contrast, the difference in shade would not be quite obvious," but then entire similarity would be.

Foil made of such gold would have the adhesive property as much as other foil when first or subsequently annealed; hence, I say, that gold having the peculiar deep color, whether Abbey's or of any other manufacturer, has not the "adhesive quality of which the doctor speaks, to all the extent that its most ardent advocate would desire;" and further, this want of adhesion is owing to the use and presence of iron.

Mr. Abbey again misconstrues me when he represents me as holding the idea "that no adhesiveness can be found in Abbey's foil *because* of this deep color." I only gave the color as a *sign* of non-adhesiveness as a *symptom*, not as a *cause*. Speaking of adhesive foil made recently by James Leslie, of Cincinnati, (misprinted Lester, in the report,) for Dr. W. M. Hunter, I said, "it may be presumed that every manufacturer of good foil can supply such an article. It will never be found to possess the deep color of Abbey's foil, as when that is present, *it cannot be made to adhere*." Such foil, if it has been kept shut up from particles floating in the atmosphere, may be re-annealed at any time before using, when it will be found to adhere with a good deal of tenacity, which, *as used* by some gentlemen, is vastly preferred for many cavities.

On adhesion, Mr. Abbey seems to row his bark both up and down stream. It is, and it aint. It will, and it wont. First it, (Abbey's fine gold,) has that adhesive quality "to all the extent its most ardent advocates would desire, although it is not to be found to an *undue* extent in our *foil* as sold." Here follows an argument drawn from his correspondence, against the use of adhesive foil, and consequently they act on the advice of their customers, and make an article *not* very adhesive. This is a virtual admission of what I say, so far as it is applicable to their foil. I do not say this is an objection to Abbey's or any other foil, it can only appear so to those who desire this quality in the foil they use. Other foils, when properly prepared, are non-adhesive when sold, (unless otherwise ordered,) but they differ from deep colored foils in this, their adhesiveness can be restored by annealing, the deep color cannot be so altered. The first will, therefore, suit both classes of operators; the other can only satisfy one.

If, in this article, I have been able to correct any erroneous impressions of my reviewers, I shall be gratified. I made no promise to do so at the commencement, if I have accomplished it, they and the profession can judge. I thank them for their efforts in my behalf and wish them better success next time. Let faithful yet courteous discussion increase, none can fear it but those who fear the light of truth.

With the best of feelings, personally, towards both reviewers and reviewed, I subscribe myself,

Truly, their servant,

A. M. LESLIE.

CINCINNATI, *August*, 1855.

